# Thermal Properties of Lightweight Steel Concrete Wall Panels under Different Humidity Conditions

**DOI:** 10.3390/ma15093193

**Published:** 2022-04-28

**Authors:** Vladimir Rybakov, Irina Ananeva, Anatoly Seliverstov, Kseniia Usanova

**Affiliations:** 1 Institute of Civil Engineering, Peter the Great St. Petersburg Polytechnic University, 195251 St. Petersburg, Russia; usanova_kyu@spbstu.ru; 2 OTSK, Ltd., 194223 St. Petersburg, Russia; irina.ananeva94@yandex.ru; 3 Sovbi, Ltd., 191024 St. Petersburg, Russia; sovbitex@mail.ru

**Keywords:** lightweight steel concrete structures, steel profile, foam concrete, thermal resistance, thermal conductivity coefficient

## Abstract

The paper presents the thermal properties of lightweight steel concrete wall panels under different humidity conditions: under normal operation conditions and high moisture of the structure. The total thermal resistance (considering thermal inhomogeneity) of the enclosing lightweight steel concrete structure with a thickness of 310 mm using monolithic low-density foam concrete (density grade of D200), at an equilibrium humidity of 5% and 8%, was experimentally established. It was equal to 4.602 m^2.0^C/W and 4.1 m^2.0^C/W, respectively. In the dry state, the total thermal resistance of this structure was 5.59 m^2.0^ C/W, which corresponds to a thermal conductivity coefficient of 0.057 m °C/W. The influence of both horizontal and vertical joints of lightweight steel concrete wall panels and the absence of thermoprofiles on thermal properties was insignificant when using heat-insulating gaskets. The actual total thermal resistance of the structure was 2.5–2.8 times higher than that obtained by calculation under high-humidity conditions (29–32%). At the same time, the decrease in the value compared to the same value at an equilibrium humidity of 5% was only 4–6%. This indicates the good workability even of a structure with high-humidity foam concrete if the reduced total thermal resistance is complied with by the standardized one.

## 1. Introduction

Lightweight steel concrete structures consist of a steel profile (profile steel frame) with a monolithic low-density foam concrete and panel sheathing [1]. 

The use of these structures in various buildings is possible in the form of slabs, and external (bearing and enclosing) and internal walls. In such structures, monolithic foam concrete is used as a heat-insulating material, which does not have the disadvantages of the main traditional ones [2,3,4].

Monolithic foam concrete is a noncombustible material [5], has a low thermal conductivity from 0.07 to 0.20 W/(m⋅°C), and has a closed porosity [6,7]. The thermal conductivity of foam concrete can be reduced to 0.062 W/(m⋅°C) by introducing perlite, basalt and glass fiber, wollastonite, and diopside [8,9,10,11]. In addition, the use of monolithic foam concrete as thermal insulation can reduce the operational energy needs of the building, which will lead to a compromise between embodied and operational energy [12]. The disadvantages of foam concrete include significant shrinkage [13]. It worsens the porous structure of foam concrete and, as a result, the strength decreases and the thermal conductivity increases [14]. Different modifiers such as wollastonite, diopside, perlite, and fly ash can be used to ensure a stable cellular structure [15,16,17].

The authors of [18] described five methods for measuring construction elements’ thermal transmittance and thermal behavior. They were the heat flow meter; guarded hot plate; hot box, considering the protected hot box and the calibrated hot box; and infrared thermography. A paper [19] showed two other new methods: Representative Points Method and Weighted Area Method. In addition, there is a simplified method of calculating thermal transmittance (U-values) in light steel framing suitable for incorporating into U-value calculation software [20].

The authors of [21] found that a secondary wood stud can mitigate the thermal bridging effect of the steel frame and improve the light gauge steel frame walls’ thermal performance, which is more noticeable when there is no thermal insulation. It is also possible to reduce the negative impact from thermal bridges in lightweight steel-framed construction elements by using thermal break strips, slotted steel studs, or flange stud indentation [22].

The authors of [23] investigated the thermal performance of a modular lightweight steel framing wall, focusing on the impact of the flanking heat losses on the thermal transmittance of the wall. The calculated heat flux values varied from 22% (external surface) to +50% (internal surface) when the flanking loss was set to 0 as a reference case, and the thermal transmittance was equal to 0.30 W/(m^2^·K). The authors of [24] investigated the thermal insulation influence regarding its position in lightweight steel-framed facade walls. For the same thermal insulation thickness, differing exclusively in its position in the wall, differences between U_hom_ and U_real_ reached values of 0.38% and 94.37%. In cold construction facade walls, considering the effect of the steel stud piercing the insulation layer led to a U-value increase from 21.68% to 94.37%. The wall with a warm frame construction had the maximum computed difference between Uhom and Ureal less than 1.5%. 

Heat-protective properties of the enclosure structure from thin-wall profiles with polystyrene concrete [25], expanded polystyrene foam [26], and foamed concrete [27,28] were studied by various authors. The results [25] showed that by increasing the density of polystyrene concrete by 4.1 times (from 300 kg/m^3^ to 1300 kg/m^3^), the thermal conductivity was increased by 3.7 times. The results [26] showed that thermal insulation of the lightweight assembled exterior wall panel with expanded polystyrene foam was 0.9 W/(m^2^·K), and the same wall panel with polystyrene particle mortar was 1.15 W/(m^2^·K). The experimentally obtained value of thermal conductivity resistance of the enclosure structure from thin-wall profiles with foam concrete was 1.367 (m^2^·K)/W, considering a thermal resistance of 0.783 (m^2^·K)/W for reinforced sections [27]. In [28], the most useful density range of this material in energy saving was defined from 100 to 300 kg/m^3^.

However, there is little research on foam concrete as part of lightweight steel concrete structures. The closest is the work [27], which considered external structures with a foam concrete density from 440 to 624 kg/m^3^. Further studies are needed to determine the thermal properties of external wall panels consisting of a steel frame filled with low-density foam concrete.

The object of the study was a lightweight steel concrete wall panel.

This work aimed to study the thermal properties of lightweight steel concrete wall panels under different humidity conditions: under normal operation conditions and high moisture of the structure.

Tasks of the research:

1. Experimental determination of the thermal properties of lightweight steel concrete wall panels under normal 5% humidity conditions.
2. Determination by the calculation method of the thermal properties of lightweight steel concrete wall panels under high humidity conditions and a dry state based on the experiment.
3. Experimental determination of the thermal properties of a structure under the worst operating conditions: the presence of a horizontal field joint of two wall panels; increased structure humidity; absence of a thermoprofile in the steel frame composition.
4. The same, in the presence of a vertical field joint of panels.

## 2. Materials and Methods

### 2.1. Lightweight Steel Concrete Structures Materials

For the production of lightweight steel concrete wall panels, the following materials were used:

1. Monolithic foam concrete with D200 density grade based on Portland cement and foaming agent. Foam concrete manufactured by SOVBI Ltd. (Saint Petersburg, Russia). The SOVBI technology makes it possible to obtain nonshrinking foam concrete with a fine cellular structure that has a stable quality.
2. Sheets “Steklotsem” with thickness of 8 mm manufactured by Stroyevolyutsiya Ltd. (Moscow, Russia).
3. Galvanized steel profiles PN (channel profile) and PSt (thermoprofile) manufactured by Stal-Profil Ltd. (Saint Petersburg, Russia).

### 2.2. Research Model

**Figure 1:**
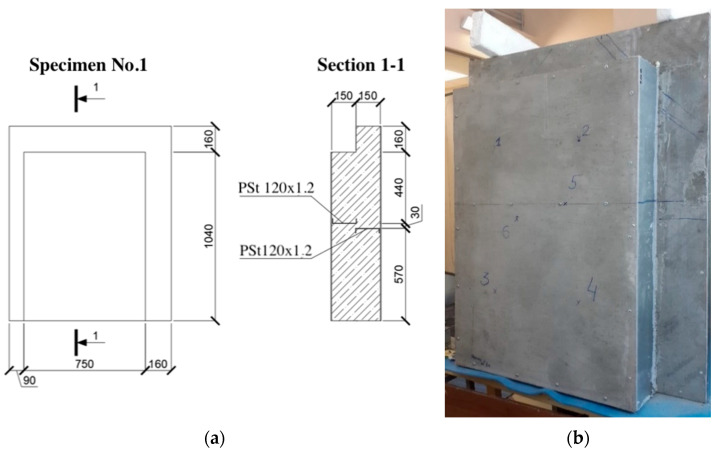
First specimen for testing. (**a**) Specimen dimensions; (**b**) specimen photo.

**Figure 2:**
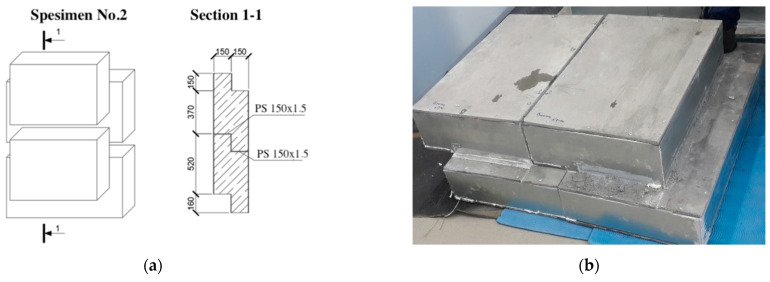
Second specimen for testing. (**a**) Specimen dimensions; (**b**) specimen photo.

**Figure 3:**
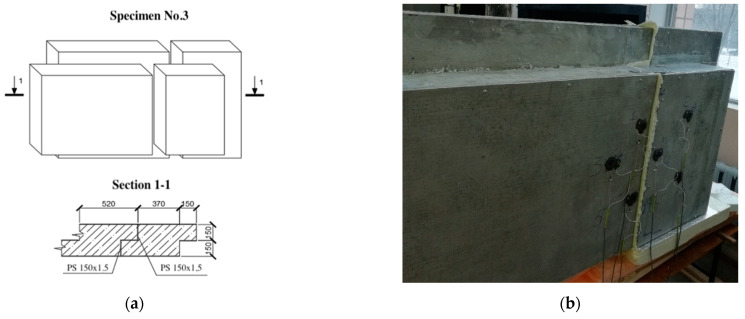
Third specimen for testing. (**a**) Specimen dimensions; (**b**) specimen photo.

The research model was represented by three specimens of lightweight steel concrete structures. The specimens consisted of steel profiles, monolithic foam concrete with D200 density grade, and “Steklotsem” sheets, which had a fixed formwork (see Figure 1, Figure 2 and Figure 3).

The first test specimen (see Figure 1) was used after 9 days of curing. The specimen had dimensions of 1000 (L) × 1200 (H) × 310 (W), where L, H, and W are the width, height, and thickness of the structure (in millimeters), respectively.

The second test specimen (see Figure 2) consisted of two panels with dimensions of 1000 (L) × 600 (H) × 310 (W), where L, H, and W are the width, height, and thickness of the structure (in millimeters), respectively. The specimen had a horizontal field joint. At the junction of the panels, two layers of heat-insulating material Izolon as a gasket were used.

The composition of specimen No. 2 was similar to that of specimen No. 1, but nonperforated profiles of PS type were used as a load-bearing framework instead of thermoprofiles of the PSt type.

The third test specimen (see Figure 3) consisted of two panels. Dimensions of the first panel were 1000 (L) × 1200 (H) × 310 (W), and those of the second panel were 1000 (L) × 600 (H) × 310 (W), where L, H, and W are the width, height, and thickness of the structure (in millimeters), respectively. The specimen had a vertical field joint. At the junction of the panels, two layers of heat-insulating material Izolon were used.

The composition of sample No. 3 was identical to that of sample No. 2, except for the dimensions of the left half-panel.

### 2.3. Methods

The input data were external temperature, internal temperature, specimen dimensions, and climatic chambers dimensions. Output data of the study were the R-value of foam concrete and R-value of foam concrete in the places of the field joint.

The tested models were placed in the design position. Further, the TX-500 climatic chamber was installed on one side, and the temperature in the cold (outer) part of the chamber was set at −30 °C. On the opposite side, there was the second heat-moisture-drying chamber SM 5/100–500; the temperature in the warm (inner) part of the chamber was set at +20 °C at a humidity of 60%. The part of the structure that did not fall into the screens of the chamber was insulated with heat-insulating materials to prevent the loss of cold and warm air from both sides of the structure.

Temperature sensors and heat flow sensors were installed on the surface of the sample. The models were tested in chambers until the state of a steady thermal process from 1 to 4 days, depending on the type of construction.

## 3. Results and Discussion

### 3.1. Determination of the Wall Panel Thermal Properties

**Figure 4:**
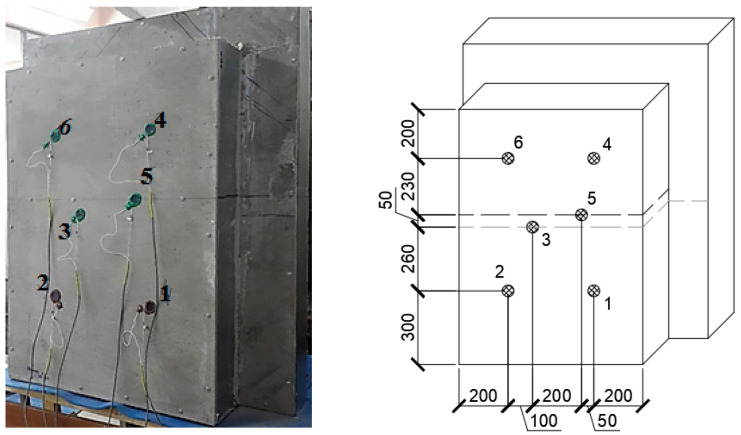
Test setup for specimen No. 1.

Specimen No. 1 with installed heat flow and temperature sensors is shown in Figure 4. Six sensors were used, two of which (No. 3, 5) were located on the steel part of the structures, and four sensors (No. 1, 2, 4, 6) were located on the foam concrete part of the structures.

The purpose of the experiment was to determine the thermal resistance of the zones of pure foam concrete and the zones of the structure part with a steel profile. 

**Figure 5:**
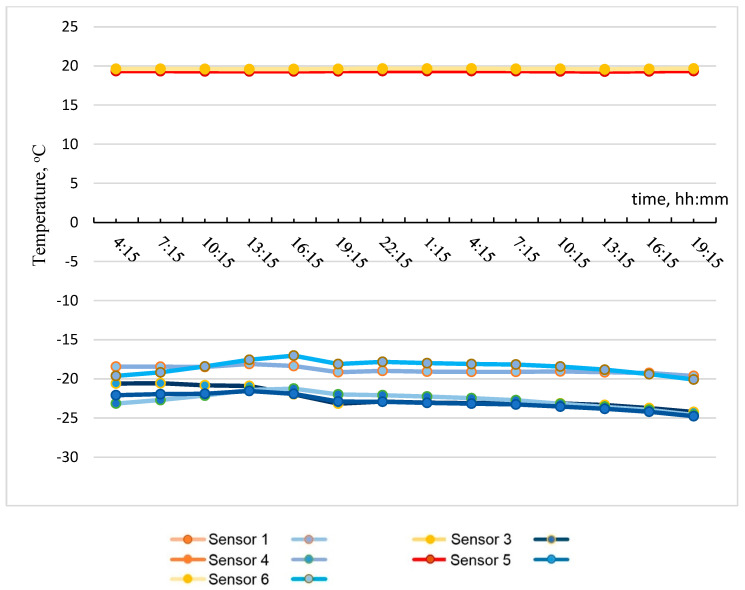
Temperature on specimen No. 1 surface with time.

**Figure 6:**
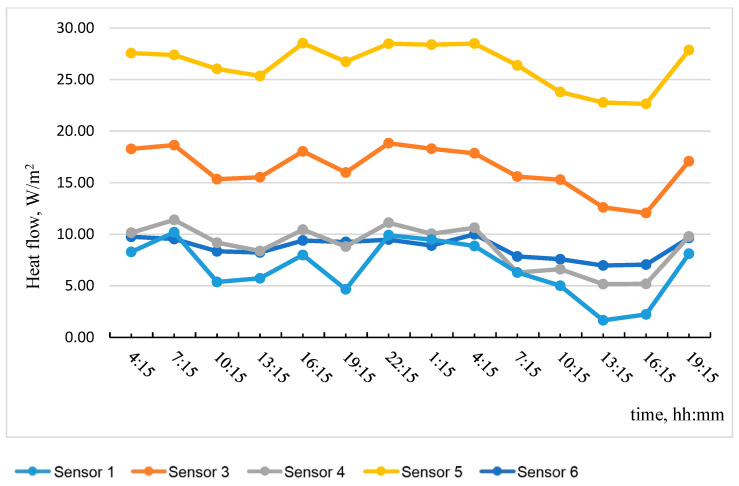
Heat flow dependence on time.

Based on the data obtained, the most temperature-stabilized estimated time intervals were selected, and graphs of temperature on the specimen No. 1 surface versus time and of heat flow versus time were plotted (see Figure 5 and Figure 6).

The apparent thermal resistance in sensors 1–5 is calculated by Formula (1):

(1) $$R=\frac{\delta }{\lambda }$$

where λ is the thermal conductivity and δ is the wall thickness.

The thermal conductivity is calculated by Formula (2):

(2) $$\lambda =\frac{q\cdot \delta }{T_{2}-T_{1}}$$

where $T_{1},T_{2}$ are wall surface temperatures and q is the heat flow.

Substituting (2) into (1), we obtain

(3) $$R=\frac{T_{2}-T_{1}}{q}\;$$

Table 1 shows the results of calculating the average apparent thermal resistance values according to the graphs in Figure 5 and Figure 6 using Formula (3), considering the zones of the tested wall fragment.

**Figure 7:**
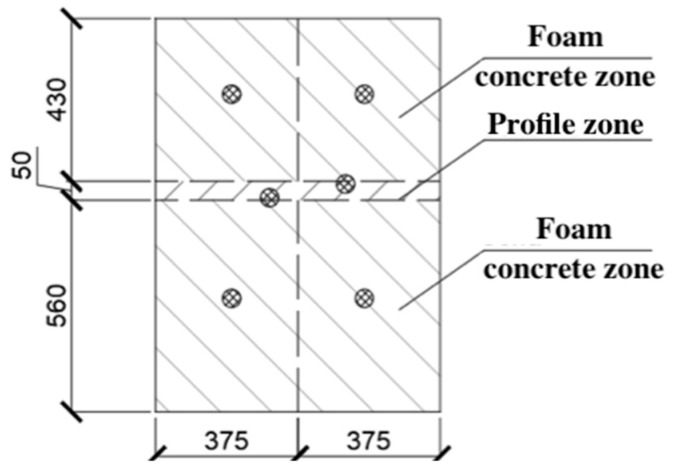
Separation of the working surface into material zones.

The total thermal resistance calculation of an inhomogeneous enclosure structure was carried out by determining the planes parallel to the direction of the heat flow. The enclosing structure (or part of it) was conditionally cut into sections. Some sections could be homogeneous (single-layer) and consist of one material, and others could be heterogeneous and consist of different materials (see Figure 7).

Table 2 shows the areas of each zone falling on the screen of the climate chamber.

The weighted average thermal resistance value *R_k_* of the tested wall fragment is calculated by Formula (4):

(4) $$R_{k}=\frac{A_{\Sigma }}{\sum_{i=1}^{n}\frac{A_{i}}{R_{i}}}=\frac{A_{\Sigma }}{\frac{A_{1}}{R_{1}}+\frac{A_{2}}{R_{2}}+\frac{A_{3}}{R_{3}}}=\frac{72.75\cdot 10^{-2}}{\frac{29.625\cdot 10^{-2}}{5.245}+\frac{3.75\cdot 10^{-2}}{2.078}+\frac{39.375\cdot 10^{-2}}{4.414}}=4.443\;\left(m^{2}\cdot K/W\right).$$

The total thermal resistance of the tested wall fragment along the surface of the wall structure $R_{T}^{}$ is calculated by Formula (5):

(5) $$R_{T}^{}=R_{si}+R_{k}+R_{se}=\frac{1}{8.7}+4.443+\frac{1}{23}=4.602\;\left(m^{2}\cdot K/W\right).$$

Thermal transmittance (according to ISO 9869-1:2014(E)) is calculated by Formula (6):

(6) $$U=\frac{1}{R_{T}^{}}=\frac{1}{4.602}=0.217\;\left(W/m^{2}\cdot K\right),$$

where $R_{si}=\frac{1}{\alpha_{int}}$ and $\alpha_{int}$ are heat transfer coefficients of the inner surfaces of the building envelope, taken for walls equal to 8.7 W/m^2^ °C according to Russian State Standard SNIP 23-02 “Thermal performance of the buildings”; $R_{se}=\frac{1}{\alpha_{ext}}\;\mathrm{and}\;\alpha_{ext}$ are heat transfer coefficients of the outer surfaces of the building envelope for cold season conditions, taken for walls equal to 23 W/m^2^ °C according to Russian State Standard SNIP 23-101 “Thermal performance design of buildings”.

For comparison, the required total thermal resistance of newly erected external walls in St. Petersburg is 3.08 m^2^·°C/W, which indicates good thermal insulation properties of monolithic foam concrete.

In this case, the average coefficient of thermal conductivity of a wall fragment with a thickness of *δ* = 0.310 mm is calculated by Formula (7):

(7) $$\lambda =\frac{\delta }{R_{k}}=\frac{0.310}{4.443}=0.07\;\left(m^{2}\cdot C^{0}/W\right).$$

It should be noted that the indicated value corresponds to the structures only at actual humidity. In practice, it is necessary to use the thermal resistances values at the normalized equilibrium moisture content of 5% and 8% and in a dry state.

### 3.2. Determination of the Reduced Total Thermal Resistance of Lightweight Steel Concrete Structures at Different Foam Concrete Moistures

#### 3.2.1. Determination of Foam Concrete Actual Moistures

The actual moisture content of foam concrete samples was determined according to Russian State Standard GOST 12730.2-2020 “Concretes. Method of determination of moisture content”. After the end of the experiment, a core with dimensions of 100 × 100 × 300 (mm) was drilled out, weighed in a wet state and placed in a low-temperature laboratory electric furnace SNOL 67/350, and dried at a temperature of +105 °C to a constant mass (after 48 h in the chamber, the mass of the sample can be considered constant). After re-weighing, the moisture content of the sample was calculated by Formula (8):

(8) $$W_{m}=\frac{m_{b}-m_{c}}{m_{c}}\cdot 100\%=\frac{651-620}{620}\cdot 100\%=5\%,$$

where $W_{m}$ is the sample moisture, %; $m_{b}$ is the sample weight before drying, g; $m_{c}$ is the sample weight after drying, g.

**Figure 8:**
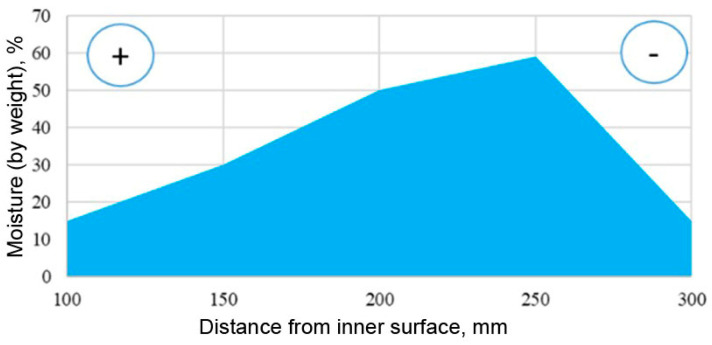
Separation of the working surface into material zones.

Figure 8 shows the moisture distribution across the wall thickness.

The graph shows that the plane of maximum masonry moisture is at a distance of 2/3 of its thickness when measured from the inner surface of the test fragment. 

#### 3.2.2. Determination of the Reduced Total Thermal Resistance at Equilibrium Moisture Content of 8%

The thermal resistance of the tested wall fragment at an equilibrium moisture of 8%, following STB EN 1745-2008, is calculated according to Formula (9):

(9) $$\begin{array}{c} R_{\mathrm{wi}}^{r}=R_{w0}^{r}\cdot e^{f_{w}\left(w_{2}-w_{1}\right)}.\\ R_{w8}^{r}=4.443\cdot e^{4\cdot \left(0.05-0.08\right)}=3.941\;\left(m^{2}\cdot C^{0}/W\right), \end{array}$$

where $f_{w}$ is a coefficient adopted following STB EN 1745-2008;

$w_{1}=8\%$ is equilibrium moisture;

$w_{2}=5\%$ is moisture of the wall fragment during the test.

Total thermal resistance is calculated by Formula (5):

$$R_{T,8}^{}=\frac{1}{8.7}+3.941+\frac{1}{23}=4.10\;\left(m^{2}\cdot K/W\right).$$

Thermal transmittance is calculated by Formula (6):

$$U_{8}^{}=\frac{1}{4.10}=0.244\;\left(W/m^{2}\cdot K\right).$$

#### 3.2.3. Determination of the Reduced Total Thermal Resistance in Dry State

The theoretical value of thermal resistance in the state at zero moisture is:

$$R_{w,\mathrm{dry}}^{r}=4.443\cdot e^{4\cdot \left(0.05-0.00\right)}=5.427\;\left(m^{2}\cdot C^{0}/W\right).$$

The average coefficient of thermal conductivity for a dry wall fragment is:

$$\lambda =\frac{\delta }{R_{w,\mathrm{dry}}^{r}}=\frac{0.310}{5.427}=0.057\;\left(m\cdot C^{0}/W\right).$$

The theoretical value of total thermal resistance in the state at zero moisture is:

$$R_{T,dry}^{}=\frac{1}{8.7}+5.427+\frac{1}{23}=5.59\;\left(m^{2}\cdot K/W\right).$$

The thermal transmittance is:

$$U_{dry}^{}=\frac{1}{5.59}=0.179\;\left(W/m^{2}\cdot K\right).$$

Next, consider the theoretical values of the reduced thermal resistance in wetting the main heat-insulating layer—foam concrete to moisture values of 29% and 32%.

#### 3.2.4. Determination of the Reduced Total Thermal Resistance under High-Humidity Conditions

The theoretical values of the reduced thermal resistance, total thermal resistance, and thermal transmittance under high-humidity conditions (29% and 32%) are, respectively:

$$R_{w29}^{r}=4.443\cdot e^{4\cdot \left(0.05-0.29\right)}=1.701\;\left(m^{2}\cdot C^{0}/W\right),$$

(10) $$R_{T,29}^{}=\frac{1}{8.7}+1.701+\frac{1}{23}=1.86\;\left(m^{2}\cdot K/W\right),$$

$$U_{29}^{}=\frac{1}{1.86}=0.538\;\left(W/m^{2}\cdot K\right),$$

$$R_{w32}^{r}=4.443\cdot e^{4\cdot \left(0.05-0.32\right)}=1.509\;\left(m^{2}\cdot C^{0}/W\right),$$

(11) $$R_{T,32}^{}=\frac{1}{8.7}+1.509+\frac{1}{23}=1.67\;\left(m^{2}\cdot K/W\right),$$

$$U_{32}^{}=\frac{1}{1.67}=0.599\;\left(W/m^{2}\cdot K\right)$$

These values are low, but they are only theoretical ones. Next, we consider an experimental study of the thermal resistance of samples at high humidity and prove their performance, considering the zones of the junction of the panels.

### 3.3. Determination of the Thermal Properties of Structure with Horizontal Joint and under High Humidity Conditions

**Figure 9:**
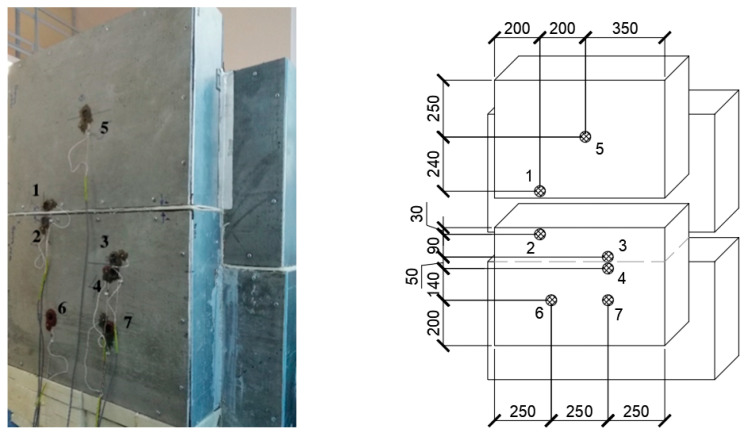
Sample No. 2 with installed thermal and temperature sensors.

Sample No. 2, which has a horizontal field joint of two wall panels with installed thermal and temperature sensors, is shown in Figure 9. Seven sensors are used, four of which are located at the field joint, and three sensors are located on the foam concrete.

**Figure 10:**
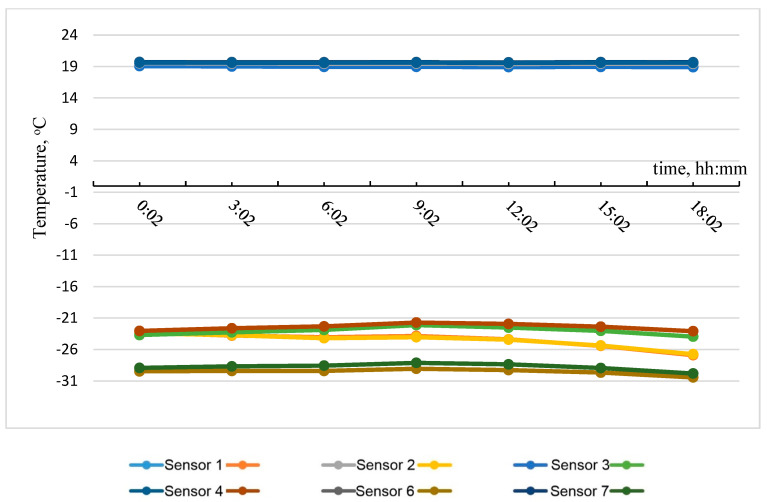
Temperature on the specimen No. 2 surface with time.

**Figure 11:**
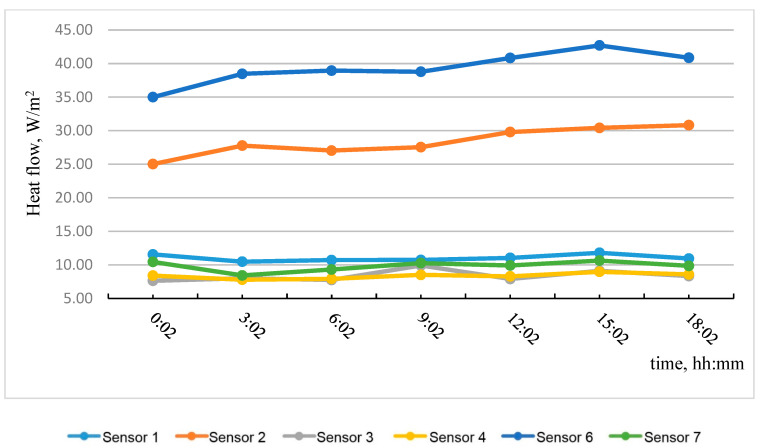
Heat flow dependence on time.

Based on the data obtained, the most temperature-stabilized estimated time intervals were selected, and graphs of temperature on the specimen No. 2 surface versus time and of heat flow versus time were plotted (see Figure 10 and Figure 11).

Table 3 shows the results of calculating the apparent thermal resistance in sensors 1–7 using Formula (3), considering the zones of the tested wall fragment.

Table 4 shows the areas of each zone falling on the screen of the climate chamber.

The weighted average thermal resistance value $R_{k}$ of the tested wall fragment is calculated by Formula (4):

$$R_{k}=\frac{70.5\cdot 10^{-2}}{\frac{39\cdot 10^{-2}}{4.879}+\frac{3.75\cdot 10^{-2}}{1.328}+\frac{27.75\cdot 10^{-2}}{4.879}}=4.272\;\left(m^{2}\cdot K/W\right).$$

The total thermal resistance of the tested wall fragment along the surface of the wall structure $R_{T}^{}$ is calculated by Formula (5):

$$R_{T}^{}=\frac{1}{8.7}+4.272+\frac{1}{23}=4.43\;\left(m^{2}\cdot K/W\right).$$

Thermal transmittance is:

$$U=\frac{1}{4.43}=0.226\;\left(W/m^{2}\cdot K\right).$$

The moisture content of the sample is:

$$W_{m}=\frac{m_{b}-m_{c}}{m_{c}}\cdot 100\%=\frac{647-455}{647}\cdot 100\%=29\%.$$

The sample moisture content of 29% does not correspond to 3–8% operating conditions. However, the total thermal resistance value, even with increased moisture of the sample, is quite high (2.5 times more than the theoretical (10)) and meets the requirements for thermal protection of buildings, for example, in St. Petersburg, which indicates the actual performance of the structure even when the foam concrete becomes wet.

### 3.4. Determination of the Thermal Properties of Structure with Vertical Joint and under High-Humidity Conditions

**Figure 12:**
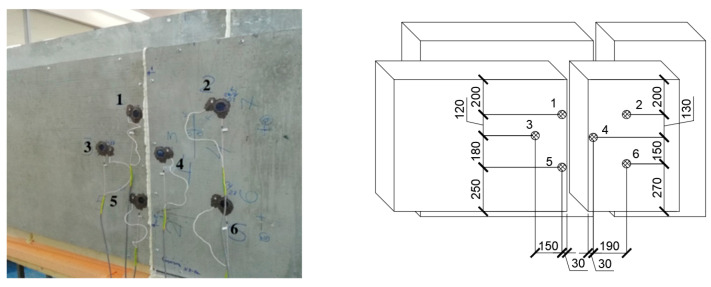
Sample No. 3 with installed thermal and temperature sensors.

Sample No. 3, which has a vertical field joint of two wall panels with installed thermal and temperature sensors, is shown in Figure 12. Six sensors are used, three of which are located at the field joint, and three sensors are located on the foam concrete.

**Figure 13:**
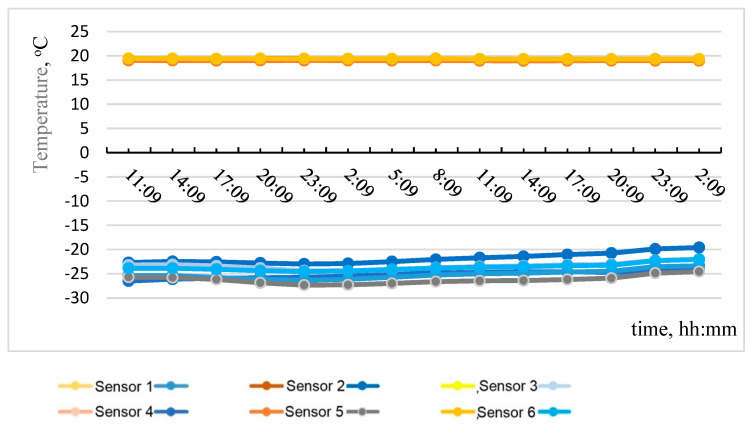
Temperature on the specimen No. 2 surface with time.

**Figure 14:**
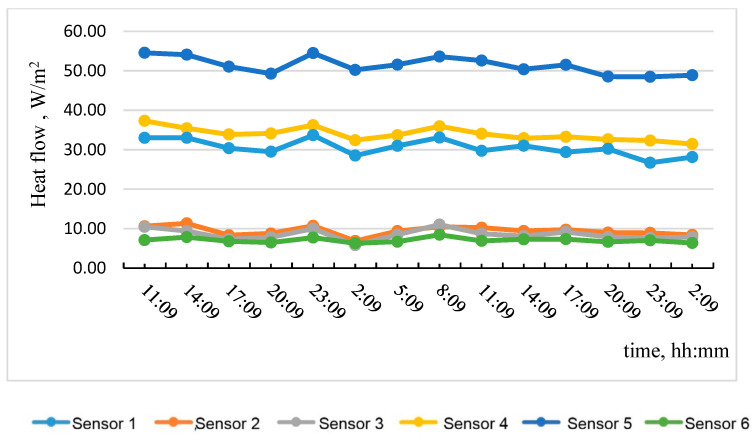
Heat flow dependence on time.

Based on the data obtained, the most temperature-stabilized estimated time intervals were selected, and graphs of temperature on the specimen No. 3 surface versus time and of heat flow versus time were plotted (see Figure 13 and Figure 14).

Table 5 shows the results of calculating the apparent thermal resistance in sensors 1–6 using Formula (3), considering the zones of the tested wall fragment.

Table 6 shows the areas of each zone falling on the screen of the climate chamber.

The weighted average thermal resistance value $R_{k}$ of the tested wall fragment is calculated by Formula (4):

$$R_{k}=\frac{60.2\cdot 10^{-2}}{\frac{28\cdot 10^{-2}}{4.995}+\frac{4.2\cdot 10^{-2}}{1.215}+\frac{28\cdot 10^{-2}}{5.216}}=4.172\;\left(m^{2}\cdot K/W\right).$$

The total thermal resistance of the tested wall fragment along the surface of the wall structure $R_{T}^{}$ is calculated by Formula (5):

$$R_{T}^{}=\frac{1}{8.7}+4.172+\frac{1}{23}=4.33\;\left(m^{2}\cdot K/W\right).$$

Thermal transmittance is:

$$U=\frac{1}{4.33}=0.231\;\left(W/m^{2}\cdot K\right),$$

The moisture content of the sample is:

$$W_{m}=\frac{m_{b}-m_{c}}{m_{c}}\cdot 100\%=\frac{966-649}{966}\cdot 100\%=32\%.$$

The sample moisture content of 32% does not correspond to 3–8% operating conditions. However, the total thermal resistance value, even with increased moisture of the sample, is quite high (2.8 times more than the theoretical (11)) and meets the requirements for thermal protection of buildings, for example, in St. Petersburg, which indicates the actual performance of the structure even when the foam concrete becomes wet.

## 4. Conclusions

The thermal properties of lightweight steel concrete wall panels were studied by experimental methods. From this research, the following conclusions can be obtained:

1. The total thermal resistance (considering thermal inhomogeneity) of the enclosing lightweight steel concrete structure with a thickness of 310 mm using monolithic low-density foam concrete (density grade of D200), at an equilibrium humidity of 5% and 8%, was established by calculation based on experimental data. It was equal to 4.602 m^2.0^ K/W and 4.1 m^2.0^ K/W, respectively, which corresponds to U-values equal to 0.217 W/m^2.0^ K and 0.244 W/m^2.0^ K, respectively.
2. In the dry state, the total thermal resistance of this structure was 5.59 m^2.0^ C/W, which corresponds to a thermal conductivity coefficient of 0.057 m °C/W and U-value equal to 0.179 W/m^2.0^ K.
3. The influence of both horizontal and vertical joints of lightweight steel concrete wall panels and the absence of thermoprofiles on thermal properties was insignificant when using heat-insulating gaskets.
4. The presence or absence of perforation in the steel profile of lightweight steel concrete wall panels did not significantly affect their thermal properties.
5. The total thermal resistance (considering thermal inhomogeneity) of the considered structure at an increased humidity of 29% and 32% was established by calculation based on experimental data. It was equal to 4.43 m^2.0^ K/W and 4.33 m^2.0^ K/W, respectively, which corresponds to U-values of 0.226 W/m^2.0^ K and 0.231 W/m^2.0^ K, respectively.
6. The actual total thermal resistance of the structure was 2.5–2.8 times higher than that obtained by calculation based on experimental data under high-humidity conditions (29–32%). At the same time, the decrease in the value compared to the same value at an equilibrium humidity of 5% was only 4–6%. This indicates good workability even of a structure with high-humidity foam concrete if the reduced total thermal resistance is complied with by the standardized one.

## Figures and Tables

**Table 1:** Fragment of a monolithic wall with a thermoprofile inside.

Fragment Zone	Inner Surface Temperature, *T*_2_ [°C]	Outer Surface Temperature, *T*_1_ [°C]	Heat Flow, *q* [W/m^2^]	Apparent Thermal Resistance, *R* [m^2^·°C/W]
Foam concrete (bottom right zone)	−18.9	19.6	8.71	4.414
Profile (zone of outer panel)	−22.5	19.5	16.39	2.56
Foam concrete (top right zone)	−22.7	19.6	8.80	4.801
Profile (zone of inner panel)	−22.9	19.9	26.46	1.597
Foam concrete (top left zone)	−18.5	19.6	6.70	5.689

**Table 2:** Areas of characteristic zones of the wall structure.

Zone	Areas of Zone [m^2^]
Foam concrete zone (area above the profile)	0.29625
Profile zone	0.0375
Foam concrete zone (area below the profile)	0.39375
Total:	0.7275

**Table 3:** Wall fragment with a horizontal field joint.

Fragment Zone	Inner Surface Temperature, *T*_2_ [°C]	Outer Surface Temperature, *T*_1_ [°C]	Heat Flow, *q* [W/m^2^]	Apparent Thermal Resistance, *R* [m^2^·°C/W]
Foam concrete (upper zone)	−26.8	19.6	8.71	5.33
Profile (zone of inner panel)	−24.6	19.3	33.86	1.328
Profile (zone of outer panel)	−22.8	19.7	8.36	5.08
Foam concrete (bottom left zone)	−29.5	19.6	11.04	4.452
Foam concrete (bottom right zone)	−28.8	19.6	9.83	4.918

**Table 4:** Areas of characteristic zones of the wall structure.

Zone	Areas of Zone [m^2^]
Foam concrete zone (area above the profile)	0.39
Profile zone	0.0375
Foam concrete zone (area below the profile)	0.2775
Total:	0.705

**Table 5:** Wall fragment with a vertical field joint.

Fragment Zone	Inner Surface Temperature, *T*_2_ [°C]	Outer Surface Temperature, *T*_1_ [°C]	Heat Flow, *q* [W/m^2^]	Apparent Thermal Resistance, *R* [m^2^·°C/W]
Foam concrete (left zone)	−23.4	19.35	8.559	4.995
Profile (left zone)	−25.39	18.99	40.543	1.095
Profile (right zone)	−25.14	19.07	33.98	1.301
Foam concrete (right zone)	−22.72	19.38	8.267	5.093
Foam concrete (left zone)	−23.4	19.35	8.559	4.995

**Table 6:** Areas of characteristic zones of the wall structure.

Zone	Areas of Zone [m^2^]
Foam concrete zone (left zone)	0.28
Profile zone	0.042
Foam concrete zone (right zone)	0.28
Total:	0.602

## Data Availability

Not applicable.
